# Comparing short‐term mortality between people with and without HIV admitted to the intensive care unit: A single‐centre matched cohort study (2000–2019)

**DOI:** 10.1111/hiv.13737

**Published:** 2024-11-20

**Authors:** N. Bakewell, T. Kanitkar, O. Dissanayake, M. Symonds, S. Rimmer, A. Adlakha, M. C. Lipman, S. Bhagani, B. Agarwal, R. F. Miller, C. A. Sabin

**Affiliations:** ^1^ Institute for Global Health University College London London UK; ^2^ National Institute for Health and Care Research (NIHR) Health Protection Research Unit (HPRU) in Blood Borne and Sexually Transmitted Infections University College London London UK; ^3^ Intensive Care Unit Royal Free Hospital, Royal Free London NHS Foundation Trust London UK; ^4^ HIV Services Royal Free Hospital, Royal Free London NHS Foundation Trust London UK; ^5^ UCL Respiratory, Division of Medicine University College London London UK; ^6^ Respiratory Medicine Royal Free Hospital, Royal Free London NHS Foundation Trust London UK; ^7^ Centre for Clinical Research in Infection and Sexual Health, Institute for Global Health University College London London UK

**Keywords:** HIV, hospital, ICU, intensive care, matched cohort study, mortality

## Abstract

**Objectives:**

The survival rate of people with HIV admitted to intensive care units (ICUs) is approaching that of people without HIV. We conducted a matched‐cohort study of people with and without HIV admitted to ICU at a large hospital to compare short‐term mortality, during 2000–2019.

**Methods:**

People with HIV were matched to people without HIV (1:2) on age, sex, admission year and Acute Physiology and Chronic Health Evaluation (APACHE)‐II score. Applying logistic regression models fitted using independence estimating equations, we describe population‐averaged associations of HIV with short‐term (in‐ICU, in‐hospital) mortality during a patient's first admission to ICU, and explore whether these varied by year.

**Results:**

A total of 177 people with HIV were matched to 354 people without HIV (71.2% vs. 71.2% male; median age: 47 vs. 48 years, median APACHE‐II: 18 vs. 17, median admission year: 2013 vs. 2013). Among people with HIV, 73.4% were on antiretroviral therapy, 51.2% had HIV‐RNA ≤50 copies/mL and median CD4 T‐cell count was 132 cells/μL. People with HIV had higher in‐ICU (24.3% vs. 15.3%) and in‐hospital (31.6% vs. 20.1%) mortality. People with HIV had 1.69‐fold higher odds (95% confidence interval: 1.03–2.76) of in‐ICU mortality and 1.86 (1.19–2.91) higher odds of in‐hospital mortality than people without HIV, adjusted for age, sex, year and APACHE‐II. There was no evidence that these associations varied by year (*p*‐interaction‐in‐ICU = 0.90; *p*‐interaction‐in‐hospital = 0.46).

**Conclusions:**

Our findings suggest that although outcomes have improved over time, people with HIV continue to have higher short‐term in‐ICU and in‐hospital mortality following ICU admission compared with people without HIV with similar characteristics.

## INTRODUCTION

The widespread use of antiretroviral therapy (ART) and advances in medical care have dramatically changed the demographic and clinical composition of the critically ill patient population of people with HIV admitted to the intensive care unit (ICU). This group now comprises an older patient population with a sustained rise in admission diagnoses due to comorbidities and conditions unrelated to HIV [1, 2, 3, 4, 5, 6, 7, 8, 9, 10]. In line with previously published studies on short‐term mortality outcomes (i.e. in‐ICU and/or in‐hospital mortality) among people with HIV admitted to ICU, we reported significant declines in short‐term mortality in a cohort of people with HIV admitted to the ICU of the Royal Free Hospital (RFH; London, UK) between 2000 and 2019 [3, 4, 5, 6, 8, 9, 10, 11]. Further, we found that important clinical factors [i.e. CD4 T‐cell count, Acute Physiology and Chronic Health Evaluation (APACHE) II score] associated with short‐term mortality in this population are unrelated to HIV, as also reported in previously published studies [3, 4, 5, 6, 8, 9, 10, 11].

Although improved survival among people with HIV admitted to ICU is evident, whether there are differences in short‐term mortality outcomes between people with and without HIV admitted to ICU remains unclear. Previous ICU‐/hospital‐based studies have reported that people with HIV have lower [7], higher [8, 9] or the same [10, 11] mortality rates while in ICU, in hospital and/or shortly after discharge as those seen in people without HIV. The incongruity in the existing evidence base may be due in part to heterogeneity in study periods, clinical practices or the type(s) of ICU. Furthermore, demographic, clinical and behavioural characteristics of people with HIV differ from those of people without HIV, but these differences were often not taken into consideration.

In this study, we aimed to compare short‐term mortality outcomes among people with and without HIV admitted to the RFH ICU between 2000 and 2019, and to assess whether the decreasing trends in short‐term mortality we previously reported for people with HIV were also seen among people without HIV.

## METHODS

### Data collection and study population

We gathered retrospective data on people with and without HIV (at least 18 years of age) admitted to the RFH ICU between 1 January 2000 and 31 December 2019. As previously described in Kanitkar et al. [4], the RFH is a large central London teaching hospital with an HIV referral centre that manages one of the largest cohorts of adults living with HIV in the UK (approximately 4000 people living with HIV, June 2020). The RFH ICU is a combined medical and surgical 34‐bed unit, which manages approximately 2000 admissions per year [4]. The data extraction procedures and sources have been previously described for people with HIV in our cohort [4, 12]; similar procedures were used to extract data for the people without HIV.

### Short‐term mortality outcomes

Short‐term mortality outcomes were in‐ICU and in‐hospital mortality at a patient's index admission (i.e. first admission) to our ICU. These were defined as death occurring prior to ICU or hospital discharge, respectively, with those dying in‐ICU also considered to have died in‐hospital.

### Variables

HIV status was the primary variable of interest. Additional variables summarized and/or included in the statistical modelling (measured at or prior to ICU admission) were age, sex, calendar year of a person's index admission to our ICU and APACHE II score. HIV‐specific variables (measured at or prior to ICU admission) summarized were CD4 count, undetectable HIV viral load (plasma HIV‐RNA ≤50 copies/mL), advanced HIV (CD4 count <200 cells/μL and/or an AIDS‐defining illness), recent known HIV diagnosis (within 3 months of ICU admission) and receipt of ART.

### Statistical analysis

People with HIV were matched to people without HIV using a 1:2 matching ratio and coarsened exact exposure‐driven matching without replacement [13]. We matched on age (<30, 30–44, 45–59, ≥60 years), sex (male/female), year (singe‐year groups, 2000–2019) and APACHE II (<15, 15–29, ≥30). People with missing data on the matching variables and/or the outcome variables were excluded prior to matching. We summarized individual's characteristics and outcomes (i.e. short‐term mortality outcomes and length of ICU stay) using medians [interquartile ranges (IQRs)] for continuous variables and counts (percentages) for categorical variables. Descriptive characteristics are provided for those with and without HIV in the full matched cohort, as well as in the full group of people who were eligible for matching. Descriptive characteristics are also provided for those with and without complete data for matching.

Regarding short‐term mortality outcomes, plots of the observed crude proportions of short‐term mortality outcomes by HIV status and admission year categorized into 4‐year periods are presented before and after matching. The Woolf test was conducted for both in‐ICU and in‐hospital mortality in the overall unmatched cohort before excluding those with missing data to assess whether the observed crude trends in short‐term mortality across the calendar periods differed by HIV status before matching and adjustment for measured potential confounders. For the primary analyses, logistic regression models were fitted using independence estimating equations (IEEs) to estimate population‐averaged odds ratios (ORs) and to obtain valid standard errors that account for matching [14]. The logistic regression models included HIV status and were further adjusted for age and APACHE II in their continuous forms to correct for any residual imbalances [14]. The primary association of interest was the main term of HIV status, but we also assessed whether trends in short‐term mortality over the 20‐year period differed by HIV status through the inclusion of an interaction term. Wald tests were used to assess statistical significance. We present plots of population‐averaged probabilities of short‐term mortality over the 20‐year period by HIV status predicted using the estimates of the final models.

### Sensitivity analyses

As matching can result in a smaller sample, reducing study power [15], we also fitted the same models in the full unmatched dataset after excluding those with missing data on matching and outcome variables and adjusting for age, sex, year and APACHE II. To assess the sensitivity of results to missing data, we refitted the matched IEE models after multiple imputation (MI) under a missing at random assumption with 50 imputed datasets.

As estimated ORs might exaggerate the magnitude of the association between HIV status and mortality outcomes (which are not rare outcomes) [16], we estimated population‐averaged risk ratios (RRs) for the associations of HIV status using the predicted population‐averaged probabilities for short‐term mortality in the final logistic regression models in the primary matched analysis only and 95% confidence intervals (CIs) were obtained using the bootstrap with 1000 bootstrap replicates [17].

All analyses were performed using R version 4.1.0, with two‐sided *p*‐values <0.05 considered to be statistically significant.

### Ethics

This project was registered as a clinical audit with RFH in July 2020, and confirmed to be an audit by RFH Research and Innovation in October 2021. All data collected were anonymized at the point of capture.

## RESULTS

In total, there were data on 20 197 index ICU admissions, 221 and 19 976 among people with and without HIV, respectively (full dataset). Of these, complete data on matching and outcome variables were available for 14 275 admissions (208 and 14 067 among people with and without HIV) that were eligible for matching (eligible dataset). In the eligible dataset, people with HIV tended to be younger (median age 46 vs. 62 years for people with and without HIV, respectively), male (71.6% vs. 57.9%), have a higher APACHE II score (median 19 vs. 16), and be admitted in earlier years of the study period (median 2012 vs. 2014). Among people with HIV, median CD4 count was 127 cells/μL, 47.4% had an undetectable HIV viral load, 72.7% were receiving ART, 24.5% were diagnosed with HIV within 3 months of their index admission to ICU, and 66.3% had advanced HIV. People with HIV had a longer ICU stay (median 5 vs. 2 days) and higher in‐ICU (27.9% vs.13.8%) and in‐hospital (37.5% vs. 18.5%) mortality (Table 1).

**TABLE 1:** Summary of patient characteristics and outcomes by HIV status for those eligible for matching (i.e. no missing data on matching and/or short‐term mortality variables), before and after matching.

*N* (%) or median (interquartile range: quartile 1–quartile 3)	Total (*N* = 14 275^a^)	People without HIV (*N* = 14 067^a^)	People with HIV (*N* = 208^a^)	Total (*N* = 531)	People without HIV (*N* = 354)	People with HIV (*N* = 177)
Age (years)	62 (49–72)	62 (49–72)	46 (38–54)	47 (40–56)	48 (39–57)	47 (40–54)
Sex at birth (male)	8289 (58.1%)	8140 (57.9%)	149 (71.6%)	378 (71.2%)	252 (71.2%)	126 (71.2%)
APACHE II	16 (12–21)	16 (12–21)	19 (14–25)	17 (12–21)	17 (12–21)	18 (13–23)
Calendar year of ICU admission^b^	2014 (2011–2017)	2014 (2011–2017)	2012 (2007–2016)	2013 (2009–2017)	2013 (2009–2017)	2013 (2009–2017)
**HIV‐specific variables**						
Blood CD4 count (cells/μL)			127 (33–302)			132 (36–319)
Missing			12			
Advanced HIV			132 (66.3%)			107 (62.9%)
Missing			9			
Undetectable			92 (47.4%)			87 (51.2%)
Missing			14			
Receipt of ART			144 (72.7%)			127 (73.4%)
Missing			10			
Recent HIV‐1 diagnosis (within 3 months of admission)			50 (24.5%)			39 (22.0%)
Missing			4			
**Clinical outcomes**						
ICU length of stay (days)	2 (1–6)	2 (1–6)	5 (2–12)	4 (2–10)	3 (2–9)	5 (2–12)
In‐ICU mortality	2006 (14.1%)	1948 (13.8%)	58 (27.9%)	97 (18.3%)	54 (15.3%)	43 (24.3%)
In‐hospital mortality	2675 (18.7%)	2597 (18.5%)	78 (37.5%)	127 (23.9%)	71 (20.1%)	56 (31.6%)

^**a**^ Patients missing data on any of the matching variables and/or outcomes were removed prior to matching, which resulted in 5922 index ICU admissions (13 and 5909 people with and without HIV, respectively) being removed from the full dataset of 20 197 index ICU admissions. Overall missingness for matching and outcome variables (number of people with missing data in parentheses): death status at ICU discharge (243), death status at hospital discharge (291), APACHE II at ICU admission (5814). Missingness overall for HIV‐specific variables (number missing before matching; number missing after matching): CD4 T‐cell count (13; 8), undetectable HIV‐RNA (16; 7), receipt of ART (12; 4), recent HIV‐1 diagnosis (6; 0).

^b^ Note, after removing participants with missing data on any of the matching factors and/or outcomes, there were no people without HIV in 2003 due to missing data on APACHE II.

We matched 177 people with HIV to 354 people without HIV (matched dataset). Within this subgroup, slight differences remained for age (median 47 vs. 48 years) and APACHE II (median 18 vs. 17). Compared with the eligible unmatched cohort, the matched cohort represented a relatively younger (median age 47 years), mostly (71.2%) male population with a moderate APACHE II (median 17) and slightly earlier median year of ICU admission of 2013. The characteristics of people with HIV in the matched cohort were similar to the eligible sample of people with HIV (Table 1). People with HIV had a longer ICU stay (median 5 vs. 3 days) and higher in‐ICU (24.3% vs. 15.3%) and in‐hospital (31.6% vs. 20.1%) mortality in this matched subgroup (Table 1).

### In‐ICU mortality

**FIGURE 1:**
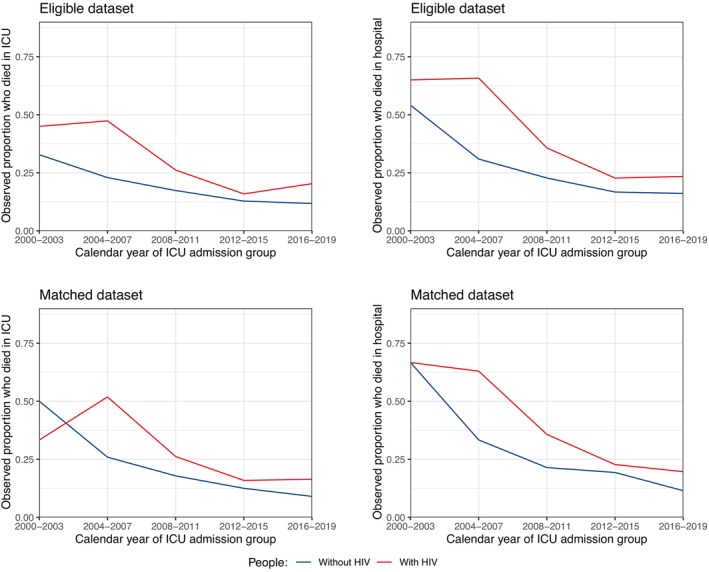
Observed proportions of intensive care unit (in‐ICU) and in‐hospital mortality by HIV status using the eligible and matched datasets.

**FIGURE 2:**
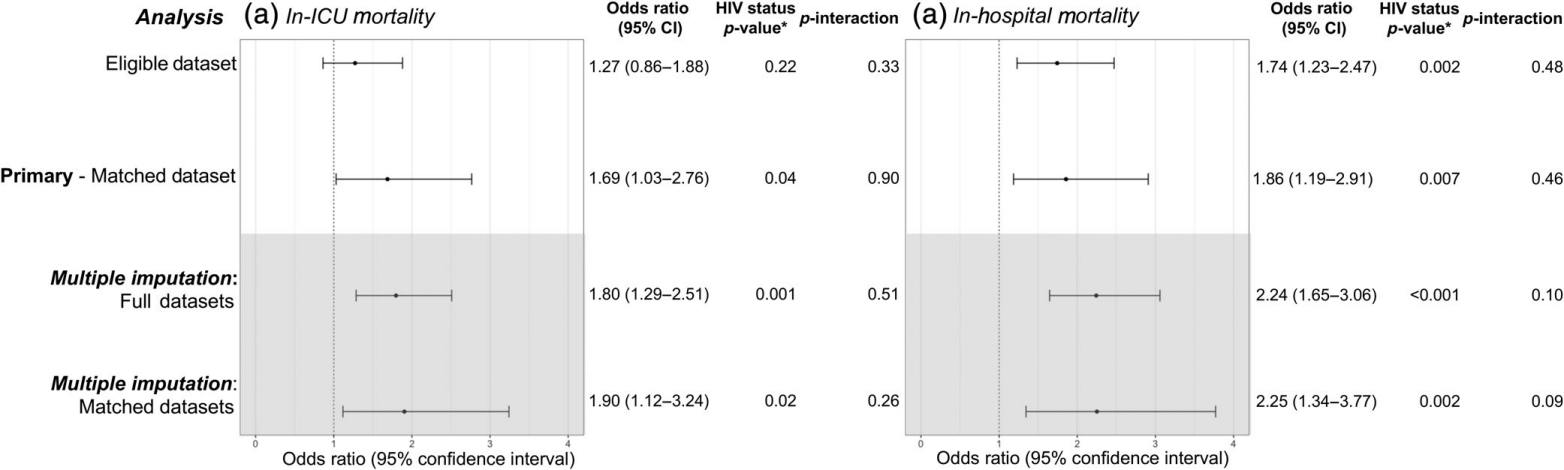
Estimated association of HIV status (referent: people without HIV), and *p*‐values for the association of HIV status and HIV status × year interaction from analyses before and after multiple imputation [odds ratios and 95% confidence intervals (CIs)]. *p‐value for the HIV status association from a model without an HIV status × calendar year interaction.

**FIGURE 3:**
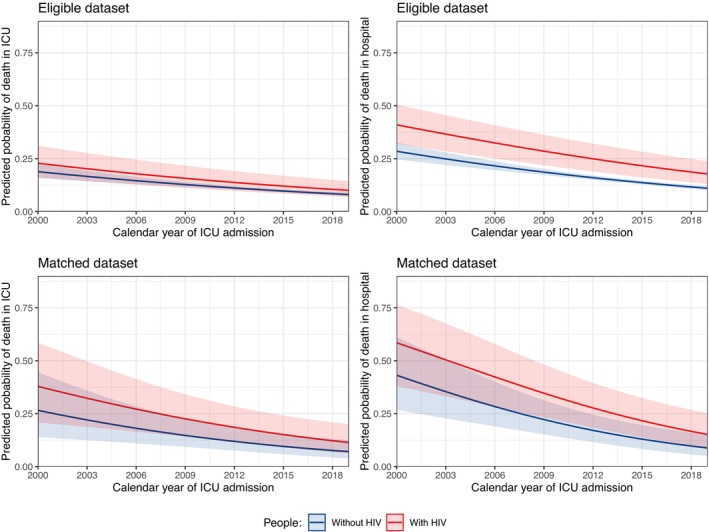
Population‐averaged predicted (using model without interaction) probabilities of intensive care unit (in‐ICU) and in‐hospital mortality by HIV status [and 95% confidence intervals (CIs)] from the logistic regressions fitted using independence estimating equations without an interaction between HIV status × year, adjusted for age, sex, year and APACHE II (single‐year increments).

Among those with data on in‐ICU mortality in the full dataset, in‐ICU mortality for people with HIV decreased from 41.7% in 2000–2003 to 20.0% in 2016–2019, and for those without HIV it decreased from 17.6% in 2000–2003 to 12.3% in 2016–2019 (Table A2). There was little evidence that crude trends in in‐ICU mortality over time differed by HIV status in this full dataset (Woolf test, *p* = 0.07). The observed calendar trends in in‐ICU mortality by HIV status in the eligible and matched datasets are presented in Figure 1. Despite a persistently higher in‐ICU mortality in those with HIV, in‐ICU mortality declined at a similar rate for both groups. People with HIV had 1.69‐fold higher odds (95% CI: 1.03–2.76) of in‐ICU mortality than people without HIV, after adjusting for age, sex, year and APACHE II, with no evidence that this association varied by year (*p*‐interaction = 0.90; Figure 2). Population‐averaged predicted probabilities of in‐ICU mortality from 2000 to 2019 by HIV status estimated using the model without an interaction are presented in Figure 3.

### In‐hospital mortality

Among those with data on in‐hospital mortality in the full dataset, in‐hospital mortality for people with HIV decreased from 58.3% in 2000–2003 to 23.1% in 2016–2019, and for those without HIV it decreased from 26.7% in 2000–2003 to 16.5% in 2016–2019 (Supplementary Material S1). In contrast to in‐ICU mortality, there was some evidence that crude trends in in‐hospital mortality over time differed by HIV status in this full dataset (Woolf test *p* = 0.01). The observed calendar trends in in‐hospital mortality by HIV status in the eligible and matched datasets are presented in Figure 1. As observed for in‐ICU mortality, people with HIV had persistently higher in‐hospital mortality than those without HIV, with similar rates of decline in this outcome over time. People with HIV had 1.86‐fold higher odds (95% CI: 1.19–2.91) of in‐hospital mortality than people without HIV, after adjusting for age, sex, year and APACHE II, with no evidence that this association varied by year (*p*‐interaction = 0.48; Figure 2). Population‐averaged predicted probabilities of in‐hospital mortality from 2000 to 2019 by HIV status estimated using the model without an interaction are presented in Figure 3.

### Sensitivity analyses

The estimated ORs were generally consistent between the primary matched analyses and sensitivity analyses using the eligible dataset before MI and the full dataset after MI with and without matching. The estimated RRs for the associations of HIV status in the matched analyses were attenuated relative to the estimated ORs [HIV status‐adjusted, population‐averaged RR (in‐ICU mortality): 1.57 (95% bootstrap CI: 1.01–2.37); HIV status‐adjusted, population‐averaged RR (in‐hospital mortality): 1.63 (1.10–2.32)], as expected given the high prevalence of the short‐term mortality outcomes over the study period.

## DISCUSSION

Our study aimed to compare short‐term mortality between people with and without HIV admitted to our ICU over the period 2000–2019. We estimated that, on average, people with HIV had 1.69‐fold and 1.86‐fold higher odds of in‐ICU and in‐hospital mortality than people without HIV, respectively, after adjusting for key confounders, and trends in these outcomes over time did not differ significantly. Therefore, despite advances in ART and intensive care management, people with HIV continue to remain at an increased risk of short‐term mortality relative to people without HIV, with similar demographic characteristics during the period 2000–2019 in our ICU cohort.

Short‐term mortality decreased for both people with and without HIV admitted to our ICU. This is consistent with declining trends in in‐ICU and in‐hospital mortality reported in the general population despite an increase in disease severity and age‐related comorbidities [18, 19]. Similar improvements in survival are documented also among people with HIV admitted to ICU [2, 5, 11, 20]. The overall in‐ICU and in‐hospital mortality in our cohort are similar to a previous UK‐based retrospective study using data collected between 1999 and 2009 which found in‐ICU and in‐hospital mortality rates of 31% and 43%, respectively, for people with HIV, compared with 20% and 26% for general medical patients in their ICU cohort [11]. Advances in both ART and intensive care strategies since the end of this previous study may help to explain the slightly lower short‐term mortality seen in people with and without HIV in our ICU cohort (using data on our cohort before missing data exclusions prior to matching). It is also important to acknowledge that, among other factors, improvements in survival among patients with and without HIV admitted to ICU and differences in short‐term mortality between ICUs/studies are also influenced by local admission and discharge policies [21].

Despite observed and estimated improvements in survival for both groups, people with HIV had persistently higher probabilities of both in‐ICU and in‐hospital mortality over the 20‐year period in our ICU cohort. This is consistent with previous population‐based reports of persistently higher short‐term mortality for people with HIV compared with demographically and/or clinically similar people without HIV [22, 23, 24]. This is also consistent with studies that compared people with and without HIV admitted to ICU reporting higher in‐ICU and in‐hospital mortality relative to people without HIV [8, 9]. By contrast, several previous ICU‐/hospital‐based studies have reported either lower [7] or no difference [10, 11, 25] in short‐term mortality for people with HIV relative to people without HIV. However, these studies often included heterogenous patient populations of people with and without HIV that were not matched or did not appropriately handle confounding using other approaches (e.g. regression adjustment) and/or similar to our cohort, making it difficult to compare our results directly. Additionally, we emphasize that one cannot infer from our data whether differences in short‐term mortality within and across ICUs/studies arise from possible health inequalities and/or inequities between these two groups. This would require future research studies to retrieve more comprehensive data on a number of measures, including critical care policies, potential HIV‐related stigma in ICU(s) and patient lifestyle and socioeconomic variables [26].

One area of research that we were not able to explore is whether there were differences in primary admission diagnoses and other measures of severity of illness and organ dysfunction, such as need for mechanical ventilation, between people with and without HIV. It has been previously reported that reasons for ICU admission are becoming similar in people with and without HIV, with most patients admitted due to acute respiratory failure and bacterial sepsis [2]. While we have included APACHE II as a measure of disease severity, it has not been prospectively validated in critically ill people with HIV; APACHE II, however, is widely accepted to be accurate in predicting ICU mortality in people with HIV in the ART era [27, 28]. In addition, we were not able to retrieve data on specific comorbidities and severity of illness and organ dysfunction factors which are key predictors of in‐ICU mortality for both people with and without HIV, such as cancer, sepsis and need for mechanical ventilation [2, 25, 29].

While we focused on short‐term mortality in general, further exploration of trends and differences in specific causes of death would be useful, particularly given the decline in HIV‐related causes of death among people with HIV admitted to ICU. Therefore, future studies should retrieve comparable and reliable data on primary admission diagnoses, states of immunosuppression, measures of severity of illness and organ dysfunction and cause of death. These can provide a more comprehensive clinical description of the patient population under study and be used to describe general short‐term mortality, specific cause of death and their trends over time, as well as assessing whether differences in trends exist between people with and without HIV for a given condition or comorbidity.

Although previous studies have compared short‐term mortality between people with and without HIV, the key strength of our study is the 20‐year period, which to our knowledge has not been considered by previous ICU‐/hospital‐based studies comparing short‐term mortality between people with and without HIV. Another key strength is our use of analyses that appropriately accounted for matching in our inferences and was further bolstered by sensitivity analyses that accounted for missing data.

However, we must acknowledge important limitations to our study. Most importantly, matching on age, sex, year and APACHE II was probably not sufficient to fully account for confounding; as such, our results may only be interpreted as associational rather than causal and should not be used alone to guide policy‐making or clinical decision‐making. There are important unmeasured confounders that future studies should consider, such as ethnicity/race and socioeconomic status [30, 31, 32]. Furthermore, other factors may also have changed over the 20‐year period, including clinical and non‐protocolized ICU admission practices and/or methods of data collection, which may have introduced sampling bias or affected the accuracy of the retrospective data sources used [12]. While our models considered a linear trend of the associations of in‐ICU and in‐hospital mortality outcomes over time in order to ensure interpretability, a non‐linear term for calendar year may be preferable. Although we have previously reported data on primary ICU admission diagnoses for the people with HIV in our study [4], we were not able to retrieve comparable data for the group of people without HIV. In assessing changes in the associations of HIV status with ICU outcomes, we recognize that our ‘exposure’ (HIV status) captures not only the direct biological effects of infection (e.g. immunosuppression, exposure to viraemia) but also possible consequences of ART and management strategies. Lastly, this was a single‐centre study conducted in the ICU of a large hospital that is experienced in HIV care; with a relatively small sample size accounted for in the primary matched analysis, and a small sample size of people with HIV overall, this limited both the power and generalizability of the results to other ICU populations of people with and without HIV.

## CONCLUSION

In conclusion, despite similar declining trends in short‐term mortality for both people with and without HIV admitted to our ICU, as well as advances in both ART and intensive care management, people with HIV had persistently higher probabilities of short‐term mortality relative to people without HIV between 2000 and 2019 in our ICU cohort. It is hoped that our robust analyses will motivate continued epidemiological and clinical research in this area. We also hope that researchers will conduct similar observational studies, appropriately controlling for confounding, to generate a more comparable evidence base in order to better inform intensive care strategies. Moreover, although data were not retrievable from additional ICUs for this study, future studies should explore the possibility of including data from multiple ICUs, with a similar 20‐year or longer study period.

## CONFLICT OF INTEREST STATEMENT

CAS has received funding from Gilead Sciences, ViiV Heathcare and Janssen‐Cilag for membership of Advisory Boards and preparation of educational materials. All other authors report no conflicts of interest.

## AUTHOR CONTRIBUTORS

BA, TK, RFM and CAS conceptualized this study. OD, TK, RFM, MS and SR directly accessed and abstracted data, with TK and RFM collating the datasets. TK and RFM verified the underlying data. NB, TK, RFM and CAS analysed the database with critical input from AA, BA, SB, OD, MCL, MS, OS and SR. NB carried out the formal statistical analyses under the supervision of CAS and wrote the original draft of the manuscript with TK, RFM and CAS. All authors provided feedback and contributed to reviewing, editing and approving the final manuscript for submission.

## Supporting information


**Data S1.** Supplementary material.

## Data Availability

The data used in this study cannot be shared publicly as they are protected health data; the information is personal or special category personal data, and there is risk of ‘re‐identification’ of data that have been pseudonymized. Access to protected health data is subject to robust governance protocols, where it is lawful, ethical and safe to do. Access to protected health data is always strictly controlled using legally binding data‐sharing contracts.

## References

[1] Finocchio T , Coolidge W , Johnson T . The ART of antiretroviral therapy in critically ill patients with HIV. J Intensive Care Med. 2019;34(11–12):897‐909. 30309292 10.1177/0885066618803871

[2] Barbier F , Mer M , Szychowiak P , et al. Management of HIV‐infected patients in the intensive care unit. Intensive Care Med. 2020;46(2):329‐342. 32016535 10.1007/s00134-020-05945-3 PMC7095039

[3] Sowah LA , George N , Doll M , et al. Predictors of in‐hospital mortality in a cohort of people living with HIV (PLHIV) admitted to an academic medical intensive care unit from 2009 to 2014: a retrospective cohort study. Medicine. 2022;101(28):e29750. 35839058 10.1097/MD.0000000000029750 PMC11132374

[4] Kanitkar T , Dissanayake O , Bakewell N , et al. Changes in short‐term (in‐ICU and in‐hospital) mortality following intensive care unit admission in adults living with HIV: 2000–2019. Aids. 2023;37(14):2169‐2177. 37605448 10.1097/QAD.0000000000003683 PMC10621640

[5] Coquet I , Pavie J , Palmer P , et al. Survival trends in critically ill HIV‐infected patients in the highly active antiretroviral therapy era. Crit Care. 2010;14(3):1‐9. 10.1186/cc9056 PMC2911753 20534139

[6] Barbier F , Roux A , Canet E , et al. Temporal trends in critical events complicating HIV infection: 1999–2010 multicentre cohort study in France. Intensive Care Med. 2014;40(12):1906‐1915. 25236542 10.1007/s00134-014-3481-7

[7] Monnet X , Vidal‐Petiot E , Osman D , et al. Critical care management and outcome of severe pneumocystis pneumonia in patients with and without HIV infection. Crit Care. 2008;12(1):1‐9. 10.1186/cc6806 PMC2374632 18304356

[8] Pathak V , Rendon ISH , Atrash S , et al. Comparing outcomes of HIV versus non‐HIV patients requiring mechanical ventilation. Clin Med Res. 2012;10(2):57‐64. 22031477 10.3121/cmr.2011.987 PMC3355737

[9] Medrano J , Álvaro‐Meca A , Boyer A , Jiménez‐Sousa MA , Resino S . Mortality of patients infected with HIV in the intensive care unit (2005 through 2010): significant role of chronic hepatitis C and severe sepsis. Crit Care. 2014;18(4):1‐9. 10.1186/s13054-014-0475-3 PMC4176576 25159592

[10] Wiewel MA , Huson MA , van Vught LA , et al. Impact of HIV infection on the presentation, outcome and host response in patients admitted to the intensive care unit with sepsis; a case control study. Crit Care. 2016;20(1):1‐10. 27719675 10.1186/s13054-016-1469-0 PMC5056483

[11] Adlakha A , Pavlou M , Walker D , et al. Survival of HIV‐infected patients admitted to the intensive care unit in the era of highly active antiretroviral therapy. Int J STD AIDS. 2011;22(9):498‐504. 21890545 10.1258/ijsa.2011.010496

[12] Bakewell N , Kanitkar T , Dissanayake O , et al. Estimating the risk of mortality attributable to recent late HIV diagnosis following admission to the intensive care unit: a single‐centre observational cohort study. HIV Med. 2022;23:1163‐1172. 36404292 10.1111/hiv.13436 PMC10099479

[13] Iacus SM , King G , Porro G . Causal inference without balance checking: coarsened exact matching. Political Anal. 2012;20(1):1‐24.

[14] Sjölander A , Johansson AL , Lundholm C , Altman D , Almqvist C , Pawitan Y . Analysis of 1: 1 matched cohort studies and twin studies, with binary exposures and binary outcomes. Stat Sci. 2012;27:395‐411.

[15] Sjölander A , Greenland S . Ignoring the matching variables in cohort studies–when is it valid and why? Stat Med. 2013;32(27):4696‐4708. 23761197 10.1002/sim.5879

[16] Davies HTO , Crombie IK , Tavakoli M . When can odds ratios mislead? BMJ. 1998;316(7136):989‐991. 9550961 10.1136/bmj.316.7136.989 PMC1112884

[17] Austin PC . Absolute risk reductions, relative risks, relative risk reductions, and numbers needed to treat can be obtained from a logistic regression model. J Clin Epidemiol. 2010;63(1):2‐6. 19230611 10.1016/j.jclinepi.2008.11.004

[18] Do Yeun Kim MHL , Lee SY , Yang BR , Kim HA . Survival rates following medical intensive care unit admission from 2003 to 2013: an observational study based on a representative population‐based sample cohort of Korean patients. Medicine. 2019;98(37):e17090. 31517831 10.1097/MD.0000000000017090 PMC6750348

[19] Zimmerman JE , Kramer AA , Knaus WA . Changes in hospital mortality for United States intensive care unit admissions from 1988 to 2012. Crit Care. 2013;17(2):1‐9. 10.1186/cc12695 PMC4057290 23622086

[20] Morris A , Creasman J , Turner J , Luce JM , Wachter RM , Huang L . Intensive care of human immunodeficiency virus–infected patients during the era of highly active antiretroviral therapy. Am J Respir Crit Care Med. 2002;166(3):262‐267. 12153955 10.1164/rccm.2111025

[21] Dijkema LM , Dieperink W , van Meurs M , Zijlstra JG . Preventable mortality evaluation in the ICU. Crit Care. 2012;16(2):1‐6. 10.1186/cc11212 PMC3681346 22546292

[22] Eyawo O , Franco‐Villalobos C , Hull MW , et al. Changes in mortality rates and causes of death in a population‐based cohort of persons living with and without HIV from 1996 to 2012. BMC Infect Dis. 2017;17(1):1‐15. 28241797 10.1186/s12879-017-2254-7 PMC5329918

[23] Marcus JL , Leyden WA , Alexeeff SE , et al. Comparison of overall and comorbidity‐free life expectancy between insured adults with and without HIV infection, 2000–2016. JAMA Netw Open. 2020;3(6):e207954. 32539152 10.1001/jamanetworkopen.2020.7954 PMC7296391

[24] Croxford S , Kitching A , Desai S , et al. Mortality and causes of death in people diagnosed with HIV in the era of highly active antiretroviral therapy compared with the general population: an analysis of a national observational cohort. Lancet Public Health. 2017;2(1):e35‐e46. 29249478 10.1016/S2468-2667(16)30020-2

[25] Akgün KM , Krishnan S , Butt AA , et al. CD4+ cell count and outcomes among HIV‐infected compared with uninfected medical ICU survivors in a national cohort. AIDS. 2021;35(14):2355‐2365. 34261095 10.1097/QAD.0000000000003019 PMC8563390

[26] Nyblade L , Stangl A , Weiss E , Ashburn K . Combating HIV stigma in health care settings: what works? J Int AIDS Soc. 2009;12(1):1‐7. 10.1186/1758-2652-12-15 PMC2731724 19660113

[27] Afessa B , Green B . Clinical course, prognostic factors, and outcome prediction for HIV patients in the ICU: the PIP (Pulmonary complications, ICU support, and prognostic factors in hospitalized patients with HIV) study. Chest. 2000;118(1):138‐145. 10893371 10.1378/chest.118.1.138

[28] Nickas G , Wachter RM . Outcomes of intensive care for patients with human immunodeficiency virus infection. Arch Intern Med. 2000;160(4):541‐547. 10695695 10.1001/archinte.160.4.541

[29] Szychowiak P , Boulain T , Timsit JF , et al. Clinical spectrum and prognostic impact of cancer in critically ill patients with HIV: a multicentre cohort study. Ann Intensive Care. 2023;13(1):74. 37608140 10.1186/s13613-023-01171-4 PMC10444715

[30] Salihu HM , Henshaw C , Salemi JL , et al. Temporal trends and black–white disparity in mortality among hospitalized persons living with HIV in the United States. Medicine. 2019;98(9):e14584. 30817575 10.1097/MD.0000000000014584 PMC6831347

[31] McGowan SK , Sarigiannis KA , Fox SC , Gottlieb MA , Chen E . Racial disparities in ICU outcomes: a systematic review. Crit Care Med. 2022;50(1):1‐20. 34636803 10.1097/CCM.0000000000005269

[32] Mullany DV , Pilcher DV , Dobson AJ . Associations between socioeconomic status, patient risk, and short‐term intensive care outcomes. Crit Care Med. 2021;49(9):e849‐e859. 34259436 10.1097/CCM.0000000000005051

