# Supplementary material for: Comparing short‐term mortality between people with and without HIV admitted to the intensive care unit: A single‐centre matched cohort study (2000–2019)
Source: HIV Med. 2024 Nov 20;26(2):275–84. doi: 10.1111/hiv.13737 (PMC11786619; doi:10.1111/hiv.13737)
Supplement: Supplementary file 1 — Data S1. Supplementary material. [file HIV-26-275-s001.docx]

# **Supplementary Material**

Comparing short-term mortality between people with and without HIV admitted to the intensive care unit: a single-centre matched cohort study (2000 to 2019)

N Bakewell^1,2^, T Kanitkar^3,4^, O Dissanayake^4^, M Symonds^4^, S Rimmer^3^, A Adlakha^3^, MC Lipman^4,5,6^, S Bhagani^4^, B Agarwal^3^, RF Miller*^4,7^ CA Sabin*^1,2^

* Joint senior authors

^1^ Institute for Global Health, University College London, London NW3 2PF, UK

^2^ National Institute for Health and Care Research (NIHR) Health Protection Research Unit (HPRU) in Blood Borne and Sexually Transmitted Infections, University College London, London NW3 2PF, UK

^3^ Intensive Care Unit, Royal Free Hospital, Royal Free London NHS Foundation Trust, London NW3 2QG, UK

^4^ HIV services, Royal Free Hospital, Royal Free London NHS Foundation Trust, London NW3 2QG, UK

^5^ UCL Respiratory, Division of Medicine, University College London, London, NW3 2PF, UK.

^6^ Respiratory Medicine, Royal Free Hospital, Royal Free London NHS Foundation Trust, London NW3 2QG, UK

^7^ Centre for Clinical Research in Infection and Sexual Health, Institute for Global Health, University College London, London WC1E 6JB, UK

| **Table A1.** Summary of patient characteristics and outcomes by those without and with missing data on matching and/or outcome variables | | | |
| --- | --- | --- | --- |
| N (%) or median (interquartile range: quartile 1, quartile 3) | *Total*  *N=20197* | *Not missing data on matching and outcome variables*  *N=14275* | *Missing data on matching and/or outcome variables*  *N=5922* |
| Age (years) | 60 (60, 72) | 62 (62, 72) | 58 (58, 71) |
| Sex at birth, male | 11763 (58.2%) | 8289 (41.9%) | 3474 (58.7%) |
| APACHE II | 16 (16, 21) | 16 (16, 21) | 17 (17, 21) |
| *Missing* | 5814 | 0 | 5814 |
| Calendar year of ICU admission | 2012 (2012, 2016) | 2014 (2014, 2017) | 2004 (2004, 2006) |
| People with HIV | 221 (1.1%) | 208 (1.5%) | 13 (0.2%) |
| **HIV-specific variables^1^** |  |  |  |
| CD4+ T-cell count (cells/μL) | 123 (123, 297) | 127 (127, 302) | 32 (32, 131) |
| *Missing* | 13 | 12 | 1 |
| Advanced HIV | 143 (67.8%) | 132 (66.3%) | 11 (91.7%) |
| *Missing* | 10 | 9 | 1 |
| Undetectable | 94 (45.9%) | 92 (47.4%) | 2 (18.2%) |
| *Missing* | 16 | 14 | 2 |
| Receipt of ART | 150 (71.8%) | 144 (72.7%) | 6 (54.5%) |
| *Missing* | 12 | 10 | 2 |
| Recent HIV-1 diagnosis (within 3 months of admission) | 54 (25.1%) | 50 (24.5%) | 4 (36.4%) |
| *Missing* | 6 | 4 | 2 |
| **Clinical outcomes** |  |  |  |
| ICU length of stay (days) | 2 (2, 6) | 2 (2, 6) | 2 (2, 6) |
| In-ICU mortality | 3019 (15.1%) | 2006 (14.1%) | 1013 (17.8%) |
| *Missing* | 243 | 0 | 243 |
| In-hospital  mortality | 4106 (20.6%) | 2675 (18.7%) | 1431 (25.4%) |
| *Missing* | 291 | 0 | 291 |
| ^1^Refers to row “People with HIV” for the sample size (“N”) of people with HIV for each column. | | | |

| **Table A2.** Observed short-term mortality overall and by HIV status for (1) the full dataset without missing data (on matching variables and/or outcomes; i.e., **eligible dataset**), (2) the **matched dataset**, and (3) the **full dataset** with missing data on matching and/or outcome variables | | | | | | | | | |
| --- | --- | --- | --- | --- | --- | --- | --- | --- | --- |
| N (%) | **Overall** | | | **People with HIV** | | | **People without HIV** | | |
|  | **Eligible Dataset** | **Matched Dataset** | **Full Dataset^1^** | **Eligible Dataset** | **Matched Dataset** | **Full Dataset** | **Eligible Dataset** | **Matched Dataset** | **Full Dataset^1^** |
| **In-ICU mortality** |  |  |  |  |  |  |  |  |  |
| 2000-2003 | 29 (35.8%) | 4 (44.4%) | 422 (17.8%) | 9 (45.0%) | 1 (33.3%) | 10 (41.7%) | 20 (32.8%) | 3 (50.0%) | 412 (17.6%) |
| 2004-2007 | 113 (25.0%) | 28 (34.6%) | 623 (17.8%) | 18 (47.4%) | 14 (51.9%) | 21 (48.8%) | 95 (22.9%) | 14 (25.9%) | 602 (17.5%) |
| 2008-2011 | 581 (17.5%) | 26 (20.6%) | 617 (18.0%) | 11 (26.2%) | 11 (26.2%) | 13 (28.9%) | 570 (17.4%) | 15 (17.9%) | 604 (17.8%) |
| 2012-2015 | 601 (12.8%) | 18 (13.6%) | 636 (13.2%) | 7 (15.9%) | 7 (15.9%) | 7 (15.9%) | 594 (12.8%) | 11 (12.5%) | 629 (13.1%) |
| 2016-2019 | 682 (11.9%) | 21 (11.5%) | 721 (12.4%) | 13 (20.3%) | 10 (16.4%) | 13 (20.0% | 669 (11.8%) | 11 (9.0%) | 708 (12.3%) |
| **Overall** | **2006 (14.1%)** | **97 (18.3%)** | **3019 (15.1%)** | **58 (27.9%)** | **43 (24.3%)** | **64 (29.0%)** | **1948 (13.8%)** | **54 (15.3%)** | **2955 (15.0%)** |
| **In-hospital mortality** |  |  |  |  |  |  |  |  |  |
| 2000-2003 | 46 (56.8%) | 6 (66.7%) | 660 (27.0%) | 13 (65.0%) | 2 (66.7%) | 14 (58.3%) | 33 (54.1%) | 4 (66.7%) | 646 (26.7%) |
| 2004-2007 | 153 (33.8%) | 35 (43.2%) | 857 (24.6%) | 25 (65.8%) | 17 (63.0%) | 28 (65.1%) | 128 (30.9%) | 18 (33.3%) | 829 (24.1%) |
| 2008-2011 | 762 (22.9%) | 33 (26.2%) | 800 (23.6%) | 15 (35.7%) | 15 (35.7%) | 18 (40.0%) | 747 (22.8%) | 18 (21.4%) | 782 (23.4%) |
| 2012-2015 | 785 (16.8%) | 27 (20.5%) | 821 (17.3%) | 10 (22.7%) | 10 (22.7%) | 10 (22.7%) | 775 (16.7%) | 17 (19.3%) | 811 (17.2%) |
| 2016-2019 | 929 (16.2%) | 26 (14.2%) | 968 (16.6%) | 15 (23.4%) | 12 (19.7%) | 15 (23.1%) | 914 (16.1%) | 14 (11.5%) | 953 (16.5%) |
| **Overall** | **2675 (18.7%)** | **127 (23.9%)** | **4106 (20.6%)** | **78 (37.5%)** | **56 (31.6%)** | **85 (38.5%)** | **2597 (18.5%)** | **71 (20.1%)** | **4021 (20.4%)** |
| ^1^Note, there are missing data for outcomes in the full dataset with missing outcomes for people without HIV. For in-ICU mortality (number missing): 2000-2003 (243), all other 4-year groups have no missing data on in-ICU mortality. For in-hospital mortality: 2000-2003 (171), 2004-2007 (5), 2008-2011 (40), 2012-2015 (73), 2016-2019 (2). | | | | | | | | | |

**Figure A1.** Population-averaged predicted (using model without interaction) probabilities of in-ICU and in-hospital mortality by HIV status (and 95% confidence intervals (CIs)) from the full and matched datasets logistic regressions analyses fitted using independence estimating equations without an interaction between HIV status and year, and further adjusted for age, sex, and APACHE II (after multiple imputation, single-year increments)
